# Impact of AI on the Cyber Kill Chain: A Systematic Review

**DOI:** 10.1016/j.heliyon.2024.e40699

**Published:** 2024-12-02

**Authors:** Mateusz Kazimierczak, Nuzaira Habib, Jonathan H. Chan, Thanyathorn Thanapattheerakul

**Affiliations:** a Division of Engineering Science, University of Toronto, Toronto, Canada; b Innovative Cognitive Computing (IC2) Research Center, School of Information Technology (SIT) King Mongkut's University of Technology Thonburi, Bangkok, Thailand

**Keywords:** Cybersecurity, Cyber attacks, Artificial intelligence in cybersecurity, Cyber kill chain, Adversarial AI, AI-based cyber attacks, Systems security, Intrusion/anomaly detection and malware mitigation, Software and application security

## Abstract

The Cyber Kill Chain (CKC) defense model aims to assist subject matter experts in planning, identifying, and executing against cyber intrusion activity, by outlining seven stages required for adversaries to execute an attack. Recent advancements in Artificial Intelligence (AI) have empowered adversaries to execute sophisticated attacks to exploit system vulnerabilities. As a result, it is essential to consider how AI-based tools change the cyber threat landscape and affect the current standard CKC model. Thus, this study examines and categorizes how attackers use AI-based tools, and offers potential defense mechanisms. We conducted a systematic literature review of 62 papers published between 2013 and 2023 from the Web of Science and Google Scholar databases. Our findings indicate that AI-based tools are used most effectively in the initial stages of cyberattacks. However, we find that current defense tools are not designed to counter these sophisticated attacks during these stages. Thus, we provide insights to 1) highlight the changing threat landscape due to AI and 2) to guide the development of cyber defense mechanisms.

## 1 Introduction

Digital data storage is being increasingly adopted in major sectors including government, healthcare, energy, and transportation. Thus, securing these systems is essential for economic stability, national security and personal safety. For example, cyberattacks can alter medical scans, disrupt power grids, and gain unauthorized control over autonomous vehicles [1], [2], [3]. The cost of global cybercrime is estimated at $600 billion per year, increasing significantly since 2014 [4]. The damage associated with the exploitation of these digital systems is expected to grow with the use of new Artificial Intelligence (AI) tools [5].

AI tools can enable unprecedented levels of automation and intelligence [6]. Offensively, AI can be used maliciously to create and disseminate misinformation. Even back in 2018, Buzzfeed, a digital media company, published an AI-generated Deepfake of the US President showcasing the capabilities of Deepfake technology that can be misused in politics [7], raising privacy and impersonation concerns. Furthermore, AI-based technology empowers attackers to process large volumes of data and automatically explore various attack methods [8], [9]. Defensively, AI automates systems, aid in processing complex data, and reduce the manpower required to operate systems or repetitive processes [10]. Therefore, AI can be leveraged to strengthen current cyber defenses.

This study presents a systematic literature review following the PRISMA guidelines. We analyze how AI-based tools are used by adversaries and their effects on each stage of the current Cyber Kill Chain (CKC) model [11]. We aim to provide insight on the following research questions:

RQ 1. What adversarial AI tools and strategies can be used in each stage of the Cyber Kill Chain?
RQ 2. What tools and strategies can be used by defenders to mitigate attacks at each stage of the Cyber Kill Chain?

This research highlights how AI transforms the threat landscape at each stage and provides guidelines for the development of subsequent defense mechanisms. Section 2 provides an overview of the CKC model. Section 3 discusses the methodology of the systematic literature review. Section 4 describes how adversaries leverage AI and possible defense strategies for each stage of the CKC. Conclusion and discussion can be found in Section 5. Section 6 aims to guide future research directions based on the insights gained.

## 2 Background and related work

### 2.1 The cyber kill chain

The Cyber Kill Chain (CKC) is a military defense framework established by Lockheed Martin in 2011, which outlines seven stages an attacker must successfully complete to achieve an operation goal [11], [12]. The framework has been widely adopted by government organizations and various industry sectors. It presents a comprehensive overview of the tasks an attacker must execute along with the necessary mitigation measures required at each stage. If the defender manages to stop the attacker at any stage of the CKC, the attack will be prevented from causing further harm to the target system [11], [12], [13]. The stages are further described in sub-sections 2.2 through 2.8.

The CKC can be used to develop effective defense mechanisms which aim to prevent an attack at a specific stage. Thus, it is important to understand how each stage of the chain is affected by advancements in AI to design robust detection models. Narrowing down the focus of this inquiry, the research questions presented in the section 1 are selected.

Existing literature on the CKC explores the details and nuances involved in each stage [14], as well as explaining the applicability of this framework to specific cybersecurity settings; for example, network defense [11], multimedia services [15] or cryptocurrencies [16]. In recent years, there has been an increase in literature regarding the implications of AI and the CKC [8]. However, there is limited work that compiles both offensive and defensive techniques used at each stage of the CKC. Thus, our research provides cybersecurity specialists with this toolbox and gives an overview on the interconnectedness of these techniques.

### 2.2 Reconnaissance

The attackers actively and/or passively gather information to select a target, recognize system vulnerabilities, and assess the system's applications and networks. Defenders continuously monitor the system for unusual activity and place an emphasis on protecting vulnerable users. This stage is typically the most time consuming, as the attackers aim to maximize their knowledge on the target system.

### 2.3 Weaponization

The weaponization stage follows reconnaissance. Attackers have gathered sufficient information on the vulnerabilities of a potential target. The attackers create software containing malicious payloads, or malware, to be delivered to the target system. If the malware is detected by defenders, they will collect any relevant artifacts of the program and analyze the different aspects of the inner workings.

Malware is often created using automated tools referred to as “Weaponizers”. The weaponization stage involves two components which are bundled together in the deliverable payload. First, a Remote Access Tool (RAT) is created to establish a C&C connection with the infected machine. The second component is the exploit, which is responsible for installing the RAT on the host machine and evading user detection [14].

The defender is tasked with gathering data on how the attackers have weaponized the delivered payload. This can be done through performing malware analysis on the artifacts left on infected computers. Understanding the information about the malware allows the defenders to find potentially unpatched exploits and generate strategies to protect systems from similar types of malware.

### 2.4 Delivery

During this stage, the malicious payload is delivered to the target system [11]. This can occur via email, website URL, a malicious USB stick, etc. Defenders aim to identify vulnerable users and information and analyze the medium through which the malicious payload was delivered.

Advancement in AI enables attackers to create more intelligent malware that evades detection by choosing an optimal attack time, adapting to defense measures, and self-learning the system environment [17], [18], [19]. The improvement of social engineering techniques increases the probability of a successful malware delivery.

Defenders should strive to detect malicious activity as it first enters the system to prevent the attacker from traversing through the chain. By leveraging machine learning techniques, the defenders can accelerate data analysis, process high volumes of data, and enhance model flexibility to better detect adversarial delivery.

### 2.5 Exploitation

During the exploitation stage, attackers take advantage of the target system's vulnerabilities to gain unauthorized access or control. Defenders increase awareness of potential cybersecurity attacks of users and conduct vulnerability and penetration testing.

If successful in this stage, the attackers will gain privileged access to the victim's machine and private data, progressing further into the CKC. The attacks can be especially dangerous if the attackers exploit zero-day vulnerabilities — vulnerabilities that have not been yet discovered by system administrators. Exploiting this type of vulnerability allows attackers to maintain undetected access to the victim's machine, while the vulnerability is not discovered.

There is limited literature on adversarial techniques during the exploitation stage. The effectiveness of this phase significantly depends on the quality of data collected during the initial stages. By understanding the system during the reconnaissance stage and crafting weapons that adeptly identify system vulnerabilities, the exploitation stage becomes the phase in which the attackers leverage this data to execute their exploits.

### 2.6 Installation

The attackers will install malware that targets a vulnerability or creates access points (backdoors, rootkits, or RATs [14]) to the compromised system. Adversaries can also install backdoors or other malicious elements into the victim's environment [11], [12]. Defenders utilize endpoint protection solutions and next-generation antivirus software to detect and block abnormal and suspicious files created in the systems.

### 2.7 Command and Control

During the Command and Control (C&C) stage, the attackers establish communication channels with the target system. This enables them to remotely control the machine, read files, and further gather information about the victim and target system. Defenders will analyze network traffic to identify abnormal network communications. Additionally, they will isolate infected systems to prevent lateral movement and actively conduct research on new C&C infrastructures used by attackers.

The communication channel used by attackers is most often established through web, email or DNS protocols. The C&C infrastructure to which the victim is connected can be hosted by the attackers or consist of a network of compromised nodes [11].

The C&C stage is a crucial step in Advanced Persistent Threat (APT) attacks, highly targeted and long-term attacks that have emerged in recent years [20]. These attacks aim to establish silent communication with the victim's network for months or even years at a time to execute mission objectives [21].

### 2.8 Actions on objectives

At this stage, attackers accomplish their goal, be it data theft, destruction of systems, or moving laterally through the network. Defenders search for indicators of malicious activity, system compromisation, and unauthorized credential usage. They also perform damage assessments of the compromised systems. Attackers have full control over the target system and thus, could execute mass and targeted attacks [14].

## 3 Methodology

### 3.1 Review process

This study aims to curate a relevant and insightful set of literature for review that addresses our research questions. The systematic literature review methodology adopted by this study is based on PRISMA International Standards [22] and is guided by the PRISMA 2020 Checklist as seen in Appendix A. The authors declare that they do not have any competing interests that could impact the review process in this literature review.

### 3.2 Search and identification of literature

The Web of Science and Google Scholar databases are queried using a list of keywords. The following keyword query is used to fetch relevant papers using the “AND” and “OR” operators: “(Artificial Intelligence AND (Cybersecurity OR Cyber Kill Chain OR Adversarial AI))”. Only studies published between 2013-2023 are considered, as the threat landscape before 2013 was much different and research at that time will not take modern cybersecurity risks into account. The sources have been last accessed before August 2023. Two reviewers screen the query outputs (There are 2,859 results from this query) and select the papers most relevant to our research questions. These reviewers work independently but are guided by the Annotation Strategy document located in Appendix B.

In the process of the review, we seek results in any domain of AI that can be applied by cybersecurity defenders or adversaries and have potential real world applications. We also gather data on the effectiveness of tools and strategies when implemented in real-world scenarios or experiments. The output of this search is then evaluated and extracted using our predetermined exclusion and inclusion criteria.

It is important to note that although most of the articles presented in the review implement their proposed systems and provide real application examples and case studies validating their findings, some articles work in the theoretical domain. The evidence from these studies may be limited since it is difficult to predict the real world behavior of the proposed systems.

### 3.3 Exclusion and inclusion criteria

The following exclusive criteria are used to determine if a study should be discarded from the set:

1. Articles that do not discuss topics relevant to the research questions (CKC, Cybersecurity models/strategies, attack models/strategies).
2. Articles that discuss non-AI attack models/strategies.
3. Articles that discuss outdated defense models/strategies. Outdated is defined as defense models which do not protect against current threats.

The following inclusive criteria are used to determine if a study should be included in the set:

1. Articles that discuss topics relevant to the research questions (CKC, Cybersecurity models/strategies, attack models/strategies).
2. Articles that either discuss AI driven attack models/strategies or defense models/strategies which consider current threats.
3. Research which uses effective validation experiments on novel models/strategies.

### 3.4 Selecting procedure

**Figure 1:**
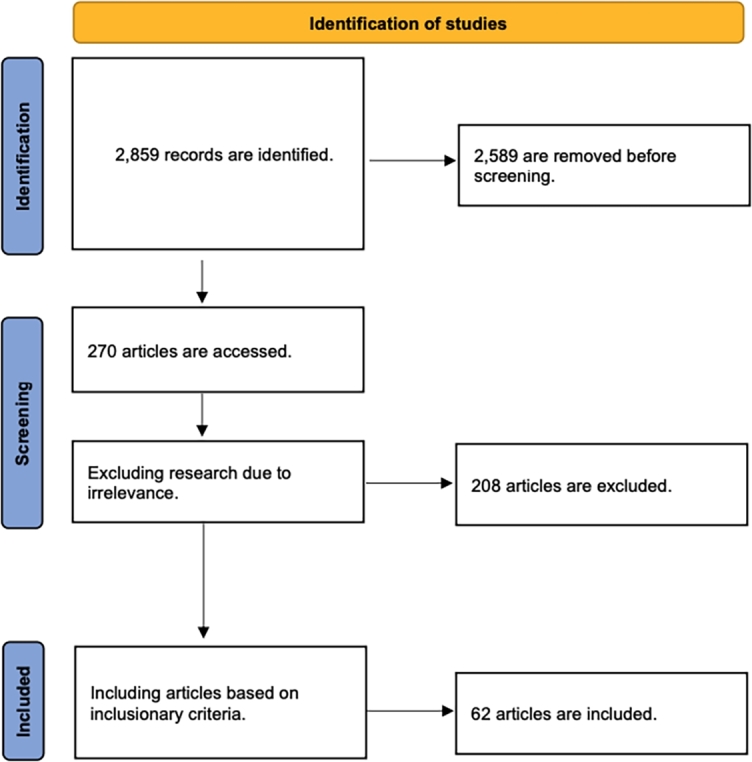
PRISMA flowchart depicting this study's review process.

Literature is searched for and identified using the Web of Science and Google Scholar databases. The PRISMA flowchart shown in Fig. 1 depicts the systematic review process used to select articles. This is conducted through the following three steps:

Step 1. **Extracting Information:** Using selected keywords, a search is conducted on the Web of Science and Google Scholar databases. This results in about 2,859 articles. Papers which are published before 2013, those with titles which indicate clear irrelevance to our research questions, and duplicates are removed before screening.
Step 2. **Screening:** The relevance of an article is determined by reviewing its content and comparing it to the predetermined exclusionary criteria. 270 research articles are manually reviewed. 120 and 150 articles are contributed by Web of Science and Google Scholar respectively. 208 are excluded due to either its non-discussion on AI driven attack models, discussion of outdated defense models, or irrelevance to this study's research questions.
Step 3. **Inclusion:** The resultant articles are assessed based on the predetermined inclusionary criteria. This study ensures that each article either discusses AI driven attacks or defense models which consider current threats and that each model is validated. A total of 62 papers are considered in this study.

### 3.5 Annotation process

The PRISMA systematic review process is used to identify 62 papers to review, these are placed in a shared Mendeley group. A flag is assigned to unannotated papers to keep track of articles that await annotation. A detailed description of the annotation strategy adopted by this study is outlined in the Annotation Strategy document given in Appendix B.

For each paper, the annotator identifies the research objective, methodology, and key findings of the investigation. The location and relevant aspects of these sections are identified in the Annotation Strategy document, located in Appendix B. These annotations are recorded on a shared Excel spreadsheet so all authors can utilize the reviewed articles to answer the research questions. Additionally, tags are assigned in the spreadsheet to indicate defense or attack tools and the specific stage to which these models are most applicable. To avoid bias, tags are given to an article by each annotator independently and compared. If these tags are conflicting, the paper must be reviewed again until the annotators reach a unanimous decision.

**Figure 2:**
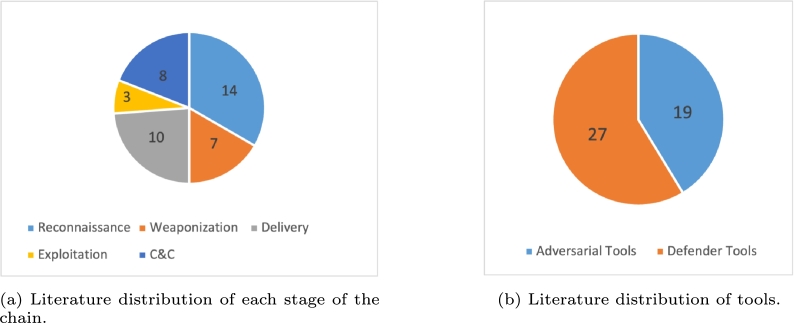
(a): Distribution of papers across the stages of the Cyber Kill Chain. (b): Distribution of papers across the types of tools.

For papers that present a strategy or tool that can be applied in real world environments and experiments, we synthesize the strategy and the specific application of it in tables at the end of each section. This study identifies 14, 7, 10, 3, and 8 papers associated with the Reconnaissance, Weaponization, Delivery, Exploitation, and C&C stages respectively, as shown in Fig. 2a. There are 20 papers which provide supplemental information and do not align with a specific stage. The selected papers contain 19 adversarial tools and 27 defender tools as seen in Fig. 2b. Some papers provide both an adversarial tool and a defender tool.

Although no quantitative inter-rater agreement between the authors was established for this study, a rigorous and qualitative process was followed to ensure that the papers selected for review were relevant to the research questions, and that the annotation strategy was consistent. The details of the inter-rater agreement process are outlined in Appendix B.

### 3.6 Narrative synthesis and data analysis

The narrative synthesis of the reviewed papers is conducted by extracting the key AI offensive and defensive strategies discussed by each paper, and the stage of the CKC they are most relevant to. As part of the data analysis, the proportion of papers presenting tools in each domain of the CKC is calculated and further discussed.

## 4 Findings and discussion

This section provides the answer to both of our RQ1. and RQ2. We discuss each stage of the CKC in more detail, including the literature of related technology and the impact of AI advancements on the stage—both from the attackers' and defenders' point of view. Each following subsection compiles literature on malicious AI tools and strategies as well as possible defense approaches to mitigate attackers at that stage. For each stage of the chain, we present a general outline of the impact of AI, and provide the most prominent examples of offensive and defensive AI techniques.

### 4.1 Reconnaissance

#### 4.1.1 Impact of AI on reconnaissance

AI has emerged as an effective tool to minimize the time needed at this stage, process large volumes of data, and create sophisticated social engineering tools. These offensive AI tools are accessible and easy to implement, enabling amateur attackers to inflict considerable damage on systems [8].

Currently, detecting adversarial reconnaissance poses as a significant challenge for defenders, partly because of its traditionally stealthy nature. A survey conducted by Mirsky et al. finds that professionals within academia, industry, and the government feel that the advancements in AI do not significantly enhance their security approaches, apart from detecting social engineering attacks [9]. This is due to the non-invasive nature of many adversarial behaviors during reconnaissance. Consequently, it is challenging to develop effective detection mechanisms.

Popularity in studying how AI aids adversaries at this stage is rising. Particularly, researchers have placed an emphasis on investigating social engineering such as phishing, deepfakes, and pretexting [8], [23], [24], [25], [26] and on how AI can aid in target selection, target identification, and information gathering [9], [14], [27].

This study finds that advancements in AI have a substantial impact on attackers during reconnaissance, largely due to the involvement of data collection at this stage. AI-powered bots and web crawlers can be used to gather large volumes of data to identify patterns, connections, and relevant information from Open Source Intelligence (OSINT), social media platforms, and websites without intervention. As a result, attackers can easily determine the optimal target to exploit during the exploitation stage, create stealthier malware in the weaponization stage, and execute effective social engineering schemes to ensure a successful delivery. Thus, with the help of automated tools, attackers can quickly traverse through the chain.

#### 4.1.2 Adversarial AI techniques

**Social Engineering**

Advancements in AI have revolutionized social engineering approaches, particularly in generating deepfakes [23], [24]. Deepfakes are synthetic media which is used to pose as someone else. GANs are emerging as a powerful tool to generate synthetic video, image, and audio content [26]. Moreover, using facial recognition algorithms, tools such as EagleEye, allow adversaries to use information imported from social media accounts to create deepfakes [25].

Mirsky et al. [9] identify 32 offensive AI tools that utilize deep learning, reinforcement learning, and natural language processing (NLP). These tools, such as point of entry detection, persona building, and target selection can significantly improve campaign planning by determining the optimal time and targets to attack. Additionally, these AI tools enhance adversarial OSINT capabilities and aid in creating sophisticated deepfakes, which are utilized in phishing attacks. By automating these planning components, inexperienced attackers can significantly increase the impact of their cyberattacks.

**Adversarial Research and Data Processing**

AI can be used to quickly obtain significant amounts of information on the system, possible targets, and defense measures. AI powered tools such as GyoiThon and Deep Exploit can be used for information gathering and automatic exploitation [25]; therefore, increasing the quality of adversarial research.

A machine learning model developed by Lee and Yim [27] demonstrate how adversaries can effectively observe keyboard inputs to steal passwords with 96% accuracy. This machine learning based model bypasses security measures by distinguishing between real and defense generated keystrokes. The paper discusses how adversaries can use the model to differentiate between real keystrokes and randomly generated keystrokes induced by defensive systems. There is limited research on defense models that employ AI to generate realistic keystrokes. Thus, this attack technique can potentially grant adversaries access to restricted information, aid in harvesting email addresses, and lead to ransomware attacks.

Shokri et al. [28] investigate how machine learning models leak information on data records used for training and develops a membership inference attack using shadow training techniques. They find that their attack method achieves an accuracy of 94% and 74% using Google's and Amazon's services respectively. By obtaining defense training data, adversaries can gain insight on detection behavior and build malicious models that evade detection, identify key system vulnerabilities, and prepare their weaponization strategies accordingly.

#### 4.1.3 Mitigation techniques

**Social Engineering**

At this stage, advancements in AI have mainly improved defense measures against social engineering. Moghimi and Varjani [29] propose a rule-based detection extension, “PhishDetector”, which uses string matching and support vector machine (SVM) algorithms to successfully detect internet banking phishing with an accuracy of 99.14%. Using NLP techniques, Sawa et al. [30] construct a model which detects social engineering in an online conversation by comparing conversation topics to a topic blocklist. The blocklist is manually set, so for this to be effective, the list must be updated often to adhere to the changing threat landscape. Tiwari et al. [31] develop a heuristic based tool called PhishSpy which can alert users to phishing URLs with a 95% accuracy rate.

As deepfakes become increasingly realistic, researchers aim to build effective safety measures. Using a Convolutional Neural Network (CNN) with a metric-learning objective function, Agarwal et al. [32] present a biometric-based deepfake detection approach that can detect face swapping. However, this method cannot identify lip-sync deepfakes. Bayar and Stamm [33] propose a CNN architecture that uses Deep Learning (DL) to detect deepfakes by identifying image manipulation features. The average accuracy of this method is found to be 99%. Researchers can also use intelligent models to filter dangerous social media posts to prevent further traversal in the chain.

Developers can also shift responsibility from cybersecurity researchers and practitioners to the user. For instance, an integrated system for the Whats-App messaging platform warns users when their messages contain hyperlinks that could lead to malicious websites [34]. This can help further safeguard users from attacks.

**Adversarial Research and Data Processing**

To ensure that attackers cannot enter the system ahead of time, defenders can ensure a strong defense model. Dreossi et al. [35] present a falsification system which uses a machine learning algorithm to assess the reliability of a defense model and indicates when more training data is required. Settanni et al. [36] presents a self-adapting anomaly detection model which can be more sensitive to adversarial reconnaissance. The first phase of this model monitors the Cyber Physical Power System (CPPS) to learn its normal behavior and better detect malicious activities in a timely manner. Yang et al. [37] propose a secure and efficient k-nearest neighbors (KNN) classification protocol using vector homomorphic encryption to secure cloud data. This algorithm ensures that attackers cannot gain access to private data.

Table 1 presents offensive techniques and corresponding defensive countermeasures developed by researchers that can be employed at the reconnaissance stage.

**Table 1:** Adversarial techniques and counterattacks adopted by adversaries and defenders during reconnaissance.

Field	Offensive technique	Relevant sources	Defensive technique	Relevant sources
**Table:** Social Engineering	**Table:** Uses generative adversarial networks (GANs) to create deepfakes.	[26]	**Table:** Uses DL to detect deepfakes by identifying image manipulation features.	[33]
	**Table:** Uses facial recognition algorithms to create the tool “EagleEve” to generate deepfakes.	[25]	**Table:** Biometric-based deepfake detection approach that can detect face swapping in images.	[32]
	**Table:** Uses “Persona Building” tools to clone or create social media profiles.	[9]	**Table:** Natural language processing (NLP) techniques to detect text based social engineering.	[30], [34]

**Table:** Adversarial research	**Table:** Tools such as GyoiThon and Deep Exploit can be used for information gathering and automatic exploitation.	[25]	**Table:** A monitoring mechanism which gathers and stores data about a system in a knowledge base from a system through sensors.	[36]
	**Table:** Observes keyboard inputs to steal sensitive passwords.	[27]	**Table:** Ensures that attackers cannot access private data using a KNN classification protocol using vector homomorphic encryption to secure cloud data.	[37]
	**Table:** Presents a “Target Selection” tool which automatically identifies the optimal victims to target in social engineering attacks	[9]		
	**Table:** Steals defense training model data to execute a membership inference attack	[28]		

### 4.2 Weaponization

#### 4.2.1 Impact of AI on weaponization

There is a lack of literature on the use of AI in offensive weaponization techniques compared to its use in defensive applications; however, there is good reason to believe that AI will change the landscape of the weaponization stage. AI significantly increases the malware's precision in target discrimination, enhancing adversarial behavior at this stage. Several past well-known cyberattacks, like the Stuxnet and Flame viruses, have infected many unexpected targets, creating collateral damage [38].

This study finds that the use of adversarial AI at this stage allows for more stealthy and better targeted victim selection. AI weaponizers can easily understand complex patterns in antivirus software and behave in a way that mimics benign software. Additionally, attackers can use powerful tools to attack only desired targets, causing little collateral damage and limiting suspicion.

#### 4.2.2 Adversarial AI techniques

**Target Discrimination**

Incorporating target selection emerges as an effective strategy for avoiding malware activation in unknown environments, thus preventing detection.

AI can and should be applied to weaponization, as discussed by C. Easttom [38]. The article gives an overview of four AI algorithms: Neural Network (NN), Decision Trees, KNN, Naïve bayes classifier, and explains how they can be applied to DL. Although several possible tasks that AI could help perform more efficiently are discussed, a special emphasis is placed on target discrimination. The authors argue that with the use of AI, not only will the attacks be more efficient by attacking a higher number of relevant targets, but it will also reduce collateral damage by minimizing unintended victims. Many novel weaponization techniques employ AI to analyze the target system and adopt an attack strategy based on the system data. This allows adversaries to understand what type of attack will yield maximal damage with minimal detection probability.

The use of AI malware to indirectly attack computing infrastructure through a cyber-physical system (CPS) can be effective because supporting CPS systems often have less developed security compared to the main systems [17]. This approach is applied to the cooling system of a supercomputer at the University of Illinois. The system first collects operational data about the CPS and learns the patterns associated with the failure of the system (e.g.: power outage, maintenance operations, emergency outage, etc.). Compared to other less intelligent methods of CPS attacks, the proposed system administers a fully targeted attack. The attack strategy is specifically tailored to disturb the normal operation of the CPS, increasing the probability of success. It also reduces the chance of detection since the attack is disguised as a CPS failure. **Stealth Malware**

Another strategy to enhance malware stealth is to automatically make small modifications as it spreads to other machines. When traditional malicious software tries to spread to other computers, it makes an exact copy of itself and sends it to new systems. Intelligent malware can make small changes to the source code of its software when spreading to new computers to evade detection. As antivirus software rapidly adapts and identifies new malware, adversaries develop adaptable malware which can mutate to evade detection. The approach of “Reactively Adaptive Malware” is presented by Hamlen et at. [39]. While traditional malware often uses random signatures, defensive tools are still able to detect patterns in these generated signatures. Reactively Adaptive Malware uses AI to learn patterns in malware detection algorithms and adapt accordingly to stay undetected. Mohan and Hamlen [40] utilize the Reactively Adaptive approach to create malware referred to as “Frankenstein”. It takes advantage of other programs installed on the host computer, and copies part of their code. This disguises their behavior as benign software and makes detection very difficult.

**Compromising Machine Learning Services**

Papernot et al. [41] present an adversarial example crafting model which is composed of two phases. First, a substitute model is trained by using data labeled by the target model. Next, the trained model is used to generate adversarial examples that are most likely to be misclassified by the target model. They find that some algorithms possess a higher transferability and efficiency between different architectures in adversarial sample crafting, but produce more noticeable perturbations in the adversarial samples. The attackers will have to find a compromise of effectiveness and stealth based on their priorities. The system can make an online MIST classifier misclassify 84% of the adversarial crafted samples. An interesting result of the research is that adversarial samples can be transferred from one machine learning algorithm to another. If the NN substitute model is trained using a specific combination of number of layers, layer size, and activation function, adversarial samples generated by this model have a high likelihood of being misclassified by a different Deep Neural Network (DNN) model with a completely different architecture.

Through review, it is evident that the development of adversarial AI tools in the domain of weaponization is currently focused on implementing intelligent environment-aware malware.

#### 4.2.3 Mitigation techniques

In defending against novel weaponization techniques, the trending approach focuses on analyzing malware execution and rewriting it to prevent the malware from executing malicious code while preserving the benign programs.

**Stealth Malware**

Wartell et al. [42] present a method that ensures that malware does not violate any security policies by automatically rewriting its program binaries. This approach does not affect the functioning of benign software, as it simply rewrites the programs. However, malicious software which relies on a security violation is rewritten such that the policy is no longer violated, which disturbs the functioning of the malware. One disadvantage of the approach is that it requires a predefined security policy, thus knowledge of what binary instructions can be malicious. This drawback can be especially critical for zero-day exploits. Manually generating security policies is burdensome, time-consuming, and mistake-prone, even when developed by experts [39].

An important trend in future cybersecurity practices will involve automated inference of unsafe patterns in binary code. Wartell et al. [43] present an approach that deals with program binaries. They propose a ‘Stir system’ which does not involve modifying the binaries of all programs to prevent security policy violations, but rather allows the application to randomize their binaries and the address locations where certain objects are stored. This makes it significantly harder for other malicious software to disrupt their normal operation. This has been proven to be effective against return-oriented programming (ROP) attacks.

**Collection and Processing of Malware Artifacts**

Traditionally, defenders rely on recording and analyzing how malware behaves to prevent an attacker's success in the weaponization stage. Recent developments in AI enable defenders to collect more detailed information on how malware works.

Two novel tools for malware analysis are explained by Severi et al. [44]. The first involves analyzing infected systems and system changes due to the virus. This approach is fast and does not require heavy resource consumption. The disadvantage of this strategy is that it can be fooled by malware employing techniques like obfuscation or packing. The second option consists of running the malware on sandbox environments and dynamically observing its behavior. While performing this type of analysis is more time and resource consuming, it allows cybersecurity specialists to collect a wider range of information about the malware. However, the limits of traditional sandboxes arise; if the defenders wish to collect more complex information about the system, this may affect the behavior of the malware. For instance, connections to external sources may time out due to the heavy load on the sandbox environment. [44] proposes “Malrec”, which allows for running the malware sample and collecting enough information to replay the system execution later. By creating a replay of the execution, defenders can collect complex information about the system without changing the behavior of the malware. A dataset of more than sixty-six thousand malware execution information is created to assess the feasibility of the system. The researchers employ this system to build a malware classification tool based on natural language features. The classifier analyzes the contents of the bytes accessed by the malware from the replay. Building a DNN classifier allows the authors to detect malware based on the natural language information with an f1 score of 94.2%. This strategy is described as too heavyweight to run in a real-time traditional sandbox environment; however, the Malrec system makes it feasible to collect these features. **Compromising Machine Learning Services**

A study conducted by Papernot et al. [41] explores a “reactive” and “proactive” defensive strategy for AI models hosted as a service. The “reactive” approach involves identifying adversarial samples, whereas the “proactive” approach requires making the model itself more robust. The authors conclude that the most promising defensive method is the “proactive” approach. This can be done by training a model with a higher dimensionality and modeling complexity. The authors also point to Distillation, which allows defense models to become more resilient to adversarial samples.

Table 2 presents offensive techniques and defensive countermeasures that can be employed at the weaponization stage.

**Table 2:** Adversarial techniques and counterattacks adopted by adversaries and defenders during weaponization.

Field	Offensive technique	Relevant sources	Defensive technique	Relevant sources
**Table:** Target discrimination of malware	**Table:** Use of NN and Decision Trees for AI target discrimination.	[38]		

**Table:** Machine Learning services	**Table:** Crafting of adversarial samples to target black-box models.	[41]	**Table:** Use of the Distillation technique to improve model resilience and increase model robustness.	[41]

Stealth Malware	**Table:** Frankenstein, an AI approach to learn from benign software patterns.	[40]	**Table:** Rewriting program binaries to prevent security policy violations.	[42]
			**Table:** Stir system: Randomizing benign software binaries to prevent malicious software from attacking it.	[43]
			**Table:** Automated generation of security policies that encompass unsafe binary patterns.	[39]

Malware Artifacts			**Table:** Use of Sandbox replays to perform deep analysis, like DNN classifiers, on malware behavior data.	[44]

### 4.3 Delivery

#### 4.3.1 Impact of AI on delivery

This study finds that advancements in AI can significantly aid in both adversarial and defensive cybersecurity approaches during delivery. This is attributed to the data collection required in both attack and defense frameworks at this stage. By collecting system data in the reconnaissance stage, malware can be automatically delivered at the most vulnerable time and location. This minimizes the time and manual involvement of the attackers, allowing amateur attackers to successfully deliver operations.

There is significant research on developing AI driven detection models which intercept the attackers at this stage. Many studies show that by leveraging AI's ability to rapidly analyze data, defenders can decrease their detection time and render their system more sensitive to abnormal activity. Currently, defenders face challenges related to securing their training data and maintaining vigilance against zero-day exploits within their systems. To detect zero-day exploits, defenders can explore the potential of AI to detect abnormal behavior within their own system that indicates weak points.

This is the first stage where the advancements in AI seem to equally benefit the attackers and defenders. AI's rapid adaptability to new attack strategies and its capability to detect unusual system behavior enhance defenders' chances of interception. Identifying zero-day exploits ahead of the attackers and safeguarding training data are key to the success of interception.

#### 4.3.2 Adversarial AI techniques

**Smart Delivery Malware**

Chung and Iyer [17] develop smart malware that monitors the target system and self-launches the operation by strategically injecting an attack at the most vulnerable time and location to inflict maximal damage. This malware filters through system failure data to effectively disguise itself as accidental failures to avoid detection. Through testing, they find that this malware adopts three unique attack strategies and effectively executes random, semi targeted, and fully targeted attacks.

**System Record Tampering**

Adversaries can use machine learning to tamper with records to disguise themselves as system updates or to obstruct evidence of delivery [9].

**Attack Frameworks**

Piplai et al. [45] develop a framework based on the Fast Gradient Sign method which demonstrates AI's power, even with limited information. The study uses the Fast Gradient Sign method to bypass a Generative Adversarial Network (GAN) based network intrusion system which is trained with adversarial examples. Although the GAN classifier yields high detection scores, this study shows that this defense mechanism is still vulnerable to sophisticated attacks. When all sensitive features of the GAN classifier are present, the attack success rate is 96% and 41% if the top 3 features of the GAN classifier are not used. Yuan et al. [46] presents an end-to-end black box attack framework called GAPGAN (Graph neural network (GNN)-based Adaptive Predictive GAN) which uses GANs to evade DL defense models designed to detect malware binaries. When used against a powerful defender model, MalConv, GAPGAN is undetected during delivery with a 100% success rate.

#### 4.3.3 Mitigation techniques

**Attack Frameworks**

Using the Competitive Markov Model, Kholidy's [47] Autonomous Response Controller (ARC) enables defenders to deliver counterattacks remotely, thereby decreasing response time and automating the response action. Timely defense methods ensure that attacks are shut down before reaching the next stage, ending the adversarial mission.

**Smart Delivery Malware**

Bekerman et al. [48] provide an end-to-end supervised detection system which analyzes network traffic to accurately detect unknown malware. Random Forest, Naïve Bayes, and J48 learning algorithms are applied to train and test the model and detect new malware families with a high accuracy. This study demonstrates that using AI increases the accuracy of network traffic analysis, achieved by using different observation resolution, cross layers and protocols features. Using anomaly detection, Settanni et al. [36] design a reliable self-adapting defense mechanism to protect CPPS (Cyber-physical production systems) using the MAPE-K (monitor- analyze-plan-execute over a knowledge base) cycle. By utilizing machine learning algorithms, the analysis phase of the cycle examines security metrics retrieved from the monitoring phase to detect malicious activity.

Alzaylaee et al. [49] showcase the effectiveness of dynamic analysis by developing a DL model, “DL-Droid”, which detects Android malware with an accuracy of 97.8% and 99.6% using only dynamic features and both dynamic and static features respectively. Similarly, Wajahat [50] proposes a lightweight and resource efficient machine learning algorithm that collects information about an Android application to make a decision on its safety. This approach is especially well suited for Internet of Things (IoT) devices, where a dynamic and adaptable system with minimal overhead is ideal in order to keep up with a dynamic environment.

Using the FP-Growth (Frequent Pattern) and Markov Logic Networks algorithms, Choi et al. [51] develop a detection mechanism which quickly identifies the deployment of metamorphic malicious code with an accuracy of 91.2%. This method takes several types of malware behavior into account and outperforms the General Bayesian Network (GBN) by 8%. However, further research is required to decrease the false positive rate which reaches 13.4% when identifying the PUP behavior type.

**Fraud**

As fraudulent activity grows, Abdallah et al. [52] conduct a survey to investigate credit card, telecommunication, healthcare insurance, automobile insurance, and online auction fraud. To prevent an attack at this stage, fraud detection systems (FDS) can be administered to detect and report fraudulent activity to the system administrator. This study identifies effective AI techniques utilized in FDS such as decision trees, NNs, hidden Markov model, fuzzy NN, Gaussian mixture, and data visualization.

Table 3 presents offensive techniques and defensive countermeasures that can be employed at the delivery stage.

**Table 3:** Adversarial techniques and counterattacks adopted by adversaries and defenders during delivery.

Field	Offensive technique	Relevant sources	Defensive technique	Relevant sources
Malware	**Table:** Development of smart malware.	[17]	**Table:** Analyzes network traffic using ML techniques to identify unknown malware.	[36], [48]
			**Table:** Detects Android malware using DL.	[49], [50]
			**Table:** Detects the deployment of metamorphic malware using the FP- Growth and Markov Logic Networks algorithms.	[51]

Record Tampering	**Table:** DL can be used to generate synthetic data.	[9]		

Attack Framework	**Table:** Uses the Fast Gradient method to bypass a GAN classifier.	[45]	**Table:** Uses the Competitive Markov Model to enable defenders to deliver counterattacks remotely.	[47]
	**Table:** Uses GANs to bypass malware detection mechanisms	[48]	**Table:** Uses ML to collect and store system data to meticulously monitor the CPS	[35]

Fraud			**Table:** Building fraud detection systems using decision trees, NN, hidden Markov model, fuzzy neural network, gaussian mixture, and data visualization.	[52]

### 4.4 Exploitation

#### 4.4.1 Impact of AI on exploitation

Recent cybersecurity literature on exploitation provides tools that make it easier to understand a system and its vulnerabilities. This is especially important as systems are becoming increasingly complicated. Both defenders and attackers can use automated tools to create attack trees, which specify all the vulnerabilities a system may have. Another strategy involves modeling attackers and defenders as actors in a simulation to learn the best strategies to “win” [53]. Real world defenders and attackers can leverage this knowledge to increase the efficiency of their operations.

#### 4.4.2 Adversarial AI techniques

**Offensive/Defensive Strategy Analysis**

Bland et al. [53] model attacker-defender interactions in a cyberattack using Petri net models. The attacker and the defender are represented as “players” using a reinforcement learning algorithm. This framework enables players to mutually learn from each other and determine the most successful and cost-efficient strategies to exploit system vulnerabilities. This type of modeling can be used by attackers to plan for optimal attacks and by defenders to find effective defense strategies. These findings will also be useful for computer system administrators, as they will be able to understand their most critical vulnerabilities.

#### 4.4.3 Mitigation techniques

**Automated Fraud Detection**

Fraud cases are increasing annually, placing a growing burden on cooperate resources. Abdallah et al. [52] examine novel Fraud Prevention Systems (FPS) and FDS which are developed to protect organizations against fraud. Traditional methods of FDS are based on rule-based systems. These methods are slow to adapt to new fraud attacks and must be constantly updated.

More recent fraud detection mechanisms make use of data mining techniques, which involve collecting data, extracting useful features, classifying transactions, and identifying patterns in the data. Newer machine learning algorithms, like NNs can be more efficient in learning data patterns and achieving higher accuracy. However, challenges like imbalance in training data, rapidly changing customer behaviors, and the requirement of real time feedback mean that there is a need for improvement in system accuracy.

**Attack Tree Generation**

Falco et al. [54] explore possible approaches attackers may take during operations associated with smart cities. They describe how public administrators often lack the expertise required to comprehend the security risk of smart cities, which can lead to catastrophic attacks. Additionally, considering the extensive network of interconnected devices and systems within a smart city, it would take excessive time and resources to enumerate all the possible attack vectors manually. This study builds on the concept of attack trees, which are designed to understand all possible causes of system failure. Nodes can be connected to each other by “AND” or “OR” logic gates to better capture the requirements of the system failure.

The authors propose a novel method of automatically creating an attack tree by leveraging AI. The trees make use of concepts like the stages of the CKC and MITRE's Common Vulnerabilities and Exposures. A case study shows that when the manually and automatically generated attack trees are both applied to a network of CCTV cameras, the automatically generated attack trees are significantly more detailed and consistent.

**Power Grid Detection Models**

Wang et al. [55] design a system to predict different states of the power grid based on its current behaviors. This includes whether it is currently under attack or if there are any physical disturbances that are affecting the normal function of the grid. The authors make use of several techniques to improve the accuracy of the model. An ensemble model with different classifiers and weights (based on the training accuracy) is used. It is also acknowledged that using DL and big data processing strategies will be a crucial research direction in the field of power grid management systems in the future.

Table 4 presents offensive techniques and defensive countermeasures that can be employed at the exploitation stage.

**Table 4:** Adversarial techniques and counterattacks adopted by adversaries and defenders during exploitation.

Field	Offensive technique	Relevant sources	Defensive technique	Relevant sources
**Table:** Attack/Defense strategies	**Table:** Offenisve strategy analysis (learning attacker model).	[53]	**Table:** Defensive strategy analysis (learning defender model).	[53]
	**Table:** Automated generation of attack trees based on a system model to choose most optimal attack path.	[54]	**Table:** Automated generation of attack trees based on a system model to develop defense countermeasures against attack paths.	[54]

CPS attacks	Power grid substation vulnerabilities	[56]	**Table:** Detection of power grid attacks using machine learning.	[55], [56]

**Table:** Fraud detection	**Table:** Automated Fraud Detection systems	[52]		

### 4.5 Installation

Our findings show that there is limited research on how advancements in AI contribute to the adversarial installation. This stage demands complex decision making and responses, which is challenging for current AI to accurately predict and automate. However, utilizing AI in the previous stages to monitor the system for optimal target selection, entry points, and injection timing enhances the success of this stage. To prevent installation, defenders can leverage AI to improve trust mechanisms and strengthen security measures.

### 4.6 Command and Control

#### 4.6.1 Impact of AI on Command and Control

Developments in AI significantly improve operations for both defenders and attackers during C&C. Unlike most previous stages, we have found that the majority of offensive strategies can be countered by defensive tools, as shown in Table 5.

**Table 5:** Adversarial techniques and counterattacks adopted by adversaries and defenders during C&C.

Field	Offensive technique	Relevant sources	Defensive technique	Relevant sources
Network Intrusion	**Table:** Botnet creation and management using AI.	[70]	**Table:** Network Traffic Analysis.	[47], [48]
	**Table:** DNN to bypass intrusion detection systems.	[45]	**Table:** Efficient network data compression and classification using self-adapting algorithms.	[66]

Domain Generation	DL Domain generation	[61]	**Table:** GAN for adversarial training of domain classifiers.	[61]

IoT			**Table:** Blockchain as a data verification strategy.	[67]
			**Table:** Trust safety mechanism.	[57], [68]

Control of machine			**Table:** DNN approach for a host-based intrusion detection system.	[69]
			**Table:** Compensation for attacks on CPS.	[59]

Recent offensive research focuses on employing AI to facilitate stealthy communications with C&C servers and in managing large-scale botnet operations. Traditionally, C&C involved one centralized server hosted by the attacker, to which all infected nodes would connect. While this is easy to manage, if the server is taken down, the C&C network will collapse. Newer approaches involve the use of decentralized networks of compromised machines, where newly infected nodes are controlled by other infected nodes. These networks are highly scalable and do not depend on the resource limitations of a single server. Additionally, the network is harder to take down, as there is a plethora of nodes to be targeted [14].

Popularity in designing C&C security approaches for IoT networks is rising in cyber defense research [57]. This is most likely due to the rapid growth of IoT networks, with more services and enterprises relying on this technology each year [58].

#### 4.6.2 Adversarial AI techniques

**Bypass of Intrusion Detection Systems**

Attackers have begun to use more advanced approaches to evade detection algorithms. For example, novel techniques can be used to disguise the C&C traffic as normal network activity. However, AI can also help defenders detect possible C&C traffic in their networks [20] and even compensate for attacks in the CPS [59].

Piplai et al. [45] demonstrate that it is feasible to use adversarial attacks to bypass GAN network intrusion detection systems, even those trained with adversarial samples. This may pose as a challenge for defenders when developing cybersecurity models.

**AI Botnet Creation and Management**

Advancements in AI significantly influence Distributed Denial of Service (DDOS) attacks. While simple DDOS tools are unable to generate massive amounts of traffic, AI has simplified the creation and maintenance of large botnets [8], [60].

**Deep Learning Domain Generation**

Pseudo-randomly generating domains using a GAN are a novel approach presented by Anderson et al. [61]. These domains can be used as endpoints for C&C architectures. Previous domain generation algorithms employ different strategies ranging from uniform distribution domains to concatenating words from the English dictionary [62]. In this study, GANs are used to design a DL-based domain generation algorithm that bypasses DL-based detectors. However, it has been also shown that the domain generated by the proposed algorithm can also bypass other machine learning based detectors. Physical systems or devices that are connected or can communicate over network protocols, such as medical devices or pacemakers, can be a common path for malicious actors to establish C&C [63].

#### 4.6.3 Mitigation techniques

While attackers can greatly benefit from the use of AI in terms of the creation and management of botnets, defenders are able to use different network traffic analysis techniques based on machine learning to detect C&C communications [64]. Network intrusion detection systems traditionally rely on rule-based algorithms, where features are manually defined. New rules must be added manually once new malware is discovered; thus, such systems adapt very slowly. Using AI, malware can be detected significantly faster compared to traditional blocklist methods [65].

**Network Traffic Analysis**

Kholidy et al. [47] study the effectiveness of machine learning models that use network traffic analysis in detecting malware. The models are trained on real network data acquired from university and corporate environments. The approach presented in the paper does not consider the content itself being transmitted over the network. The advantage of this approach is that user privacy is preserved, as well as the ability of the system to work on encrypted data. Machine learning systems for network intrusion detection can leverage the large and increasing network intrusion datasets that have become available in recent years [45].

Another challenge of developing malicious network detection is the large amount of data that can be collected from the network. Jing et al. [66] develop a machine learning system that detects DDOS attacks in networks. The first stage of the system consists in compressing the network data. The authors employ a Chinese Remainder Theorem based Reversible Sketch. This method not only efficiently compresses the data, but also recovers more information about the source of the attack once the model classifies the packet as malicious. Next, a modified Multi-chart Cumulative Sum algorithm is built to classify the packets as benign or malicious. This algorithm is designed to have self-adaptation capabilities and can detect malicious traffic independently of the network protocol used by the attackers. Then, the address of the source of the DDOS attack is recovered and added to a blocklist to mitigate the attack.

**Deep Learning Domain Generation**

Anderson et al. [61] discuss the Domain Generation Algorithm's role in strengthening and increasing the robustness of detection systems for synthetic domain generation algorithms. This research demonstrates how a GAN model can be used to create adversarial samples which can be used during the training of a detection algorithm for maliciously generated domains. This can help defenders develop more sensitive cybersecurity models.

Machado and Frohlich's [67] research targets the cybersecurity of IoT CPSs, particularly focusing on the verification of data integrity of IoT devices. They discuss how operations on IoT CPS devices are time-bounded since they must conform with the sense-decide-actuate cycle of the CPS, posing a challenge for defenders. Another challenge is the resource limitations of IoT devices; thus, it is important that integrity verification processes are designed to be energy and resource efficient [67]. It is shown that previous methods of integrity verification for IoTs put too much strain on storage capabilities, are not energy efficient, and need specialized environments. Machado and Frohlich propose a three-level blockchain architecture to overcome these problems. The proposed architecture consists in the following stages:

1. IoT (establishes a domain of trust for several nodes, which communicate with each other);
2. Fog (is responsible for fault tolerance and producing cryptographic keys that are later used for the verification of data integrity);
3. Cloud (stores the IoT data and the cryptographic keys generated by the previous level).

The effectiveness of the system is demonstrated through a series of case studies.

**Trust Safety Mechanism**

Most defense strategies can address external attacks, but are unable to detect if a node inside the network is broadcasting malicious information. Thus, Wang et al. [68] develop a fog computing based trust system which can be used in networks to verify the integrity of new data and detect malicious nodes rapidly; particularly focusing on Sensor–Cloud Systems (SCS). A two-stage trust-based hierarchical mechanism is proposed to address possible internal attacks that can occur within networks.

Similarly, Wang et al. [57] present a trust-based approach for IoT integrity verification that uses cloud and edge computing. The algorithm not only detects manipulated data and malicious nodes, but also dynamically manages the load placed on the server to increase its efficiency in processing data. The task of assessing the trust of the nodes is moved away from the resource limited IoT devices, to an edge network. The edge network, which aids the IoT network in ensuring security and efficacy is divided into two stages:

• The edge network communicates closely with the IoT nodes and ensures the security of the nodes.
• The edge platform consists of more powerful nodes, whose task is to manage the load on the IoT devices to ensure maximum efficiency, parse incoming user requests, and handle special service requirements.

**Host-based Intrusion Detection**

DNNs are an efficient approach to malware detection. This does not only apply to network activity, but also to local activity. A DNN architecture for both a network-based intrusion detection system (NIDS) and a host-based intrusion detection system (HIDS) is developed by Vinayakumar et al. [69]. The first technique is based on analyzing host network activity, while the second considers the behavior of the local environment to detect intrusions. They present an architecture based on distributed computer systems, like Hadoop Map Reduce and Apache Spark to allow the system to be highly scalable. It is also found that the proposed DNN approach is more accurate in detecting malware compared to traditional machine learning methods.

When designing robust detection systems, it must be kept in mind that while it is important to be able to detect cyberattacks, it is also important to compensate for the malicious changes to the system that the attack has created. This is especially important in CPSs, like vehicles or industrial systems.

**CPS Attach Compensation**

Farivar et al. [59] design a system to estimate and compensate for cyberattacks in CPS based on NN using the Gaussian Radial Basis Function Neural Network (GRBFNN) structure. The efficiency of the algorithm is evaluated using a simulation of a truck, which is subject to attacks or other external disturbances.

Table 5 presents offensive techniques and defensive countermeasures that can be employed at the C&C stage.

### 4.7 Action on objectives

Different adversarial objectives like CPS attacks, fraud, and misinformation discussed in previous sections can be identified at this stage. However, the actions and tools required to reach this stage occur previously in the chain; therefore, they will not be discussed here.

It is not possible to point out a specific trend in this domain, because the actions taken by the attacker and defender are highly varied based on the setting, field, and differing interpretations on what classifies as action on objectives and what is merely a means to achieve these.

AI is not particularly helpful at this stage as the decision making is often made using intuition, moral judgment, emotional intelligence, and considering ethical policies. These factors pose a challenge to the current AI and limit the effectiveness of AI tools at this stage.

However, the integration of AI tools expedites the adversary's progression through the chain, enabling attackers to reach this stage [9]. Many traditional cybersecurity approaches are unable to detect sophisticated AI based attacks [5], thus researchers must reform strategies to prevent operations from reaching completion.

The actions of the defender must be executed quickly using forensic evidence to cut off unauthorized access and prevent data exfiltration, lateral movement, and further damage [11].

## 5 Conclusion

In the past decade, advancements of technology and major world events have forced organizations and institutions to undergo a digital transformation, moving their data and resources to the cloud. This has encouraged adversaries to target these entities. Additionally, due to advancements in AI technologies, these cyberattacks have a higher success rate and can be performed on a larger scale. The consequences of cyberattacks have demonstrated that it is imperative for cybersecurity defenders to understand what novel tools are being employed by attackers, and what effective defense strategies can be implemented to defend against these cyberattacks.

Our research has revealed several insights into the impact of AI on the widely adopted framework, Cyber Kill Chain. We survey papers published between 2013 and 2023, obtained from the Web of Science and Google Scholar scientific databases. We review articles using the PRISMA framework and analyze the selected papers to compile a set of tools and strategies that give insight into emerging approaches used by adversaries. These tools range from the development of GANs that cause third-party model misclassification, to tools that learn from benign software within systems, disguising itself against antivirus software.

We address our research questions by investigating how AI affects each stage of the Cyber Kill Chain and what AI tools can be used by attackers and defenders. This study finds that although AI influences all the stages of the Cyber Kill Chain, its effects are exacerbated in the first stages of the chain: Reconnaissance, Weaponization, Delivery, and C&C. This is largely attributed to the fact that these stages deal with large amounts of data. During the reconnaissance stage, the attacker must gather intelligence about potential targets and make an informed decision. Similarly, in the delivery stage, the attackers must determine the timing and method for deploying the malicious payload. AI certainly enhances attacks by enabling attackers to rapidly process larger volumes of data, as well as automating most of the tasks involved with choosing targets and delivering payloads. We find that the Action on Objectives stage is less affected by recent developments in technology, as the stage is more dependent on social factors and the aims of the attackers.

Fortunately, cybersecurity defense tools have also improved due to advancements in AI. Defenders can take advantage of more powerful AI models to perform network classification and generate more accurate and helpful system models. However, further research is required to build robust defense models capable of protecting their model training data, identifying adaptive malware, and detecting sophisticated social engineering schemes as malicious AI rapidly progresses.

One of the main limitations of this study was the large corpus of literature on the topic of AI in cybersecurity. Although this gave the authors the opportunity to select a wide range of applicable and relevant literature, it inevitably left some parts of the literature overshadowed. A future research direction will be to make use of more quantitative tools and bibliometrics to analyze and extract the most relevant information from a wider range of literature. As Table A.6 shows, there were 2,8559 records identified in the domains of Artificial Intelligence and Cybersecurity, and although this number was greatly reduced through set selection criteria, a study aggregating more perspectives would be insightful.

## 6 Research directions

This section outlines the research directions that were identified during the study. A summary of the overall research directions is provided as follows:

• Development and study of defense strategies able to detect data collection from the adversarial side (Section 6.1).
• Study of AI strategies to generate real-looking keystrokes and simulate user input (Section 6.1),
• Methods of employing AI to make decisions to identify malware software based on its patterns of data monitoring and access (Section 6.2),
• Use of AI to bypass novel defensive tools which rewrite the binaries of programs (Section 6.2).
• Algorithms, compute units, and strategies to analyze network traffic and program activity in real-time (Section 6.3).
• Study of the effectiveness of methods which combine different AI models to monitor a system (Section 6.3).
• Use of adversarial training for Command and Control prevention mechanisms (Section 6.4).

In the subsequent subsections, we provide a more detailed discussion of research directions categorized by the stage of the CKC.

### 6.1 Reconnaissance

We find that there is limited research on defense strategies that can detect adversarial data collection, giving attackers a significant advantage at this stage. Predominantly, current research focuses on developing defense models that utilize AI to detect social engineering attempts. Although the efficacy of these models is validated through testing, many studies overlook the safeguarding of training data. Without robust security measures, adversaries can manipulate training data to yield incorrect model outputs and gain information on the model, improving their campaign planning and evading detection in the subsequent stages. In the future, defenders should build more robust monitoring systems adept at identifying adversarial data collection and focus on securing the training data of their detection models.

We also found that there is a research gap concerning attackers using a DNN to observe user keyboard input while bypassing dummy data generated by defensive software. Attackers could use AI to generate more real-looking fake data, which could confuse even the most advanced defense AI tools.

### 6.2 Weaponization

This study has identified that most adversarial tools in this stage consist in monitoring its surroundings and making decisions based on the available information. However, we found no defensive techniques countering this kind of attack. A research direction of interest will be employing AI to make decisions on whether a piece of software is malware or not based on the patterns and kind of the information about the system the program is accessing.

We find that in the weaponization stage, scientific literature focuses on defensive tools as opposed to adversarial tools. Defenders can leverage AI by rewriting program binaries in a way that complies with security policies and avoids known security vulnerabilities or by employing advanced tools to record malware behavior in a controlled environment. However, we did not find a matching development on the offensive side to overcome the progress in defense.

### 6.3 Delivery

We find a lack of literature on the effectiveness of combining various AI techniques during the delivery stage. Combining techniques like supervised learning, unsupervised learning, and reinforcement learning may enhance the detection of sophisticated attacks. This is becoming crucial, as attackers develop novel offensive techniques that require robust tools to detect and prevent a wide range of attack methods.

Real time data analysis is also an important future research topic. Being able to analyze data in real time by employing efficient algorithms and computing units will be indispensable in detecting and deferring attacks in a timely manner.

### 6.4 Command and Control

There is a lack of literature in the domain of adversarial training for defensive algorithms which aim to prevent Command and Control connections. With more data being collected on attacks and the need for more precise detection algorithms, this is an important future research direction.

## 7 Data availability statement

No special data or source code was written as part of this paper. The data used in this paper is publicly available and can be accessed through the references provided in the bibliography, or through bibliometric databases.

## CRediT authorship contribution statement

**Mateusz Kazimierczak:** Writing – review & editing, Writing – original draft, Validation, Project administration, Methodology, Investigation, Formal analysis, Data curation. **Nuzaira Habib:** Writing – original draft, Methodology, Investigation, Conceptualization. **Jonathan H. Chan:** Supervision. **Thanyathorn Thanapattheerakul:** Supervision.

## Declaration of Competing Interest

The authors declare that they have no known competing financial interests or personal relationships that could have appeared to influence the work reported in this paper.

## Figures and Tables

**Table A.6:**
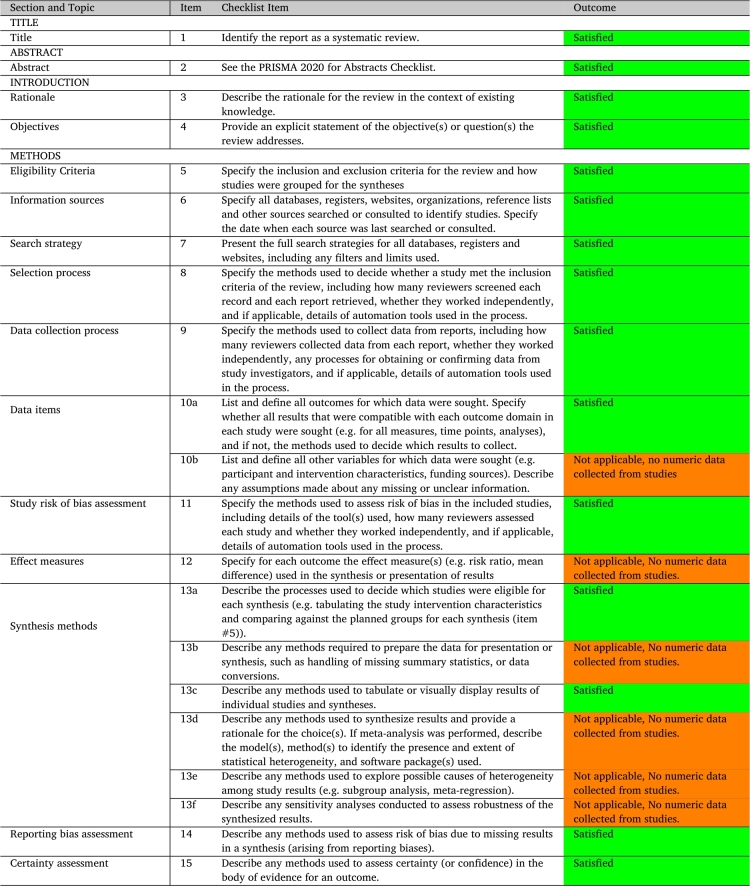
PRISMA 2020 Checklist.

**Table A.7:**
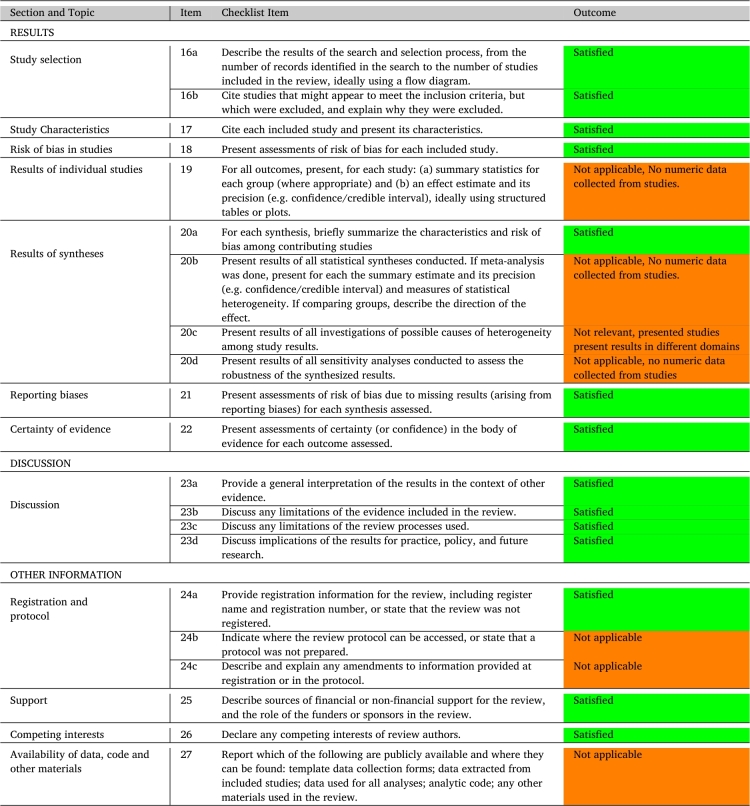
PRISMA 2020 Checklist, Cont'd.

## Appendix A PRISMA 2020 checklist

See Table A.7.

## Appendix B Annotation strategy

**Review Information**

• Our Mendeley paper repository contains 62 articles.
• The list of the papers can be found in the Mendeley repository.
• The research questions are as follows:
  RQ 1. What adversarial AI tools and strategies can be used in each stage of the Cyber Kill Chain?
  RQ 1. What tools and strategies can be used by defenders to mitigate attacks at each stage of the Cyber Kill Chain?

**Literature Selection** The agreement reached on how to select literature to review is as follows:

• The literature is retrieved from databases such as Web of Science and Google Scholar.
• The following keyword query must be utilized to fetch relevant papers using the “AND” and “OR” operators: “(Artificial Intelligence AND (Cybersecurity OR Cyber Kill Chain OR Adversarial AI))”
• Literature should be published between 2013 – 2023.
• When literature is found by an individual, it must be shared in the shared Mendeley folder so all annotators can review the research.

**Annotation**

The agreement reached on how to annotate literature during the review is as follows:

1. **Tags**
  • Using the Mendeley software add a tag indicating whether the research is most relevant to an Attacker or Defender tool. This will be indicated by the researcher within the abstract/introduction/methodology.
  • Using the Mendeley software add a tag indicating what step of the Cyber Kill Chain the paper is most relevant to. This will be determined by comparing the objective of the tool/model/framework of the research to each stage within the Lockheed Martin Cyber Kill Chain model. The set of tags that can be used in annotating the paper are: Reconnaissance, Weaponization, Delivery, Exploitation, Installation, Command and Control, and Actions on Objectives. Papers that are relevant for more than one of these stages can be tagged with multiple tags.
2. **Research Objective**
  • The research objective of this paper is to identify how each stage of the Cyber Kill Chain is affected by advancements in AI, and what are some notable offensive examples and opportunities for cybersecurity experts.
3. **Methodology**
  • Before the paper selection process, the authors have agreed on the inclusion and exclusion criteria outlined above.
  • The authors have agreed on the annotation strategy, and ensured throughout the study that their methodology was consistent with the set expectations.
  • When it was evident that the interpretation or decision selection of the paper differed among the reviewers, the paper was reviewed again, after a discussion, until a unanimous decision was reached.
